# HDGFL2 cryptic protein: a portal to detection and diagnosis in neurodegenerative disease

**DOI:** 10.1186/s13024-024-00768-y

**Published:** 2024-10-25

**Authors:** Ellen A. Albagli, Anna Calliari, Tania F. Gendron, Yong-Jie Zhang

**Affiliations:** 1 Department of Neuroscience, Mayo Clinic, Jacksonville, FL USA; 2 Neurobiology of Disease Graduate Program, Mayo Graduate School, Mayo Clinic College of Medicine, Rochester, MN USA

**Keywords:** Amyotrophic lateral sclerosis, Biomarkers, Cerebrospinal fluid, Cryptic peptide, Frontotemporal dementia, Hepatoma derived growth factor 2, Neurodegeneration, TAR DNA-binding protein 43

In 2006, TAR DNA-binding protein of 43 kDa (TDP-43) was discovered as the major ubiquitinated and aggregated protein in approximately 95% of amyotrophic lateral sclerosis (ALS) cases and 45% of frontotemporal lobar degeneration (FTLD) cases [1]. Since then, TDP-43 pathology has been identified in Alzheimer’s disease (AD), limbic-predominant age-related TDP-43 encephalopathy (LATE), and other neurodegenerative diseases [2]. This discovery initiated copious studies uncovering the pathomechanisms through which TDP-43, an RNA-binding protein with roles in alternative splicing, causes neurodegeneration [2] – chief among them, its loss of function owing to its aggregation in the cytoplasm and concurrent depletion from the nucleus.

TDP-43 proteinopathies share clinical, genetic, and pathological features, and this is particularly true of frontotemporal dementia (FTD) and ALS. While no treatments for FTD, ALS, or other TDP-43 proteinopathies yet exist, developing effective therapies for these fatal neurodegenerative diseases would benefit from biomarkers that facilitate an early and accurate diagnosis. Indeed, therapies are expected to be most effective when initiated early in the disease course. Biomarkers that identify the underlying pathology of patients with FTD in life would also aid in selecting appropriate participants for clinical trials targeting TDP-43 proteinopathy. As patients with behavioral variant FTD are essentially just as likely to develop TDP-43 or tau pathology, biomarkers that inform the presence of TDP-43 pathology would be particularly useful for this group, as would patients with AD who often develop mixed pathologies [3]. Although studies have examined whether TDP-43 itself could fulfill these biomarker needs, multiple efforts in detecting pathological TDP-43 species in biofluids have so far been unsuccessful [4]. Nevertheless, an exciting avenue being pursued harnesses the consequences of TDP-43 loss of function; more specifically, TDP-43’s inability to repress the splicing of non-conserved cryptic exons (CE) [5]. This engenders the production of novel RNA isoforms bearing non-conserved intronic sequences that often introduce frameshifts, premature stop codons, or premature polyadenylation sequences. For example, inclusion of a CE in *STMN2* mRNA produces a truncated stathmin-2 protein at the expense of its full-length counterpart, whereas inclusion of a CE in *UNC13A* mRNA reduces UNC13A protein expression (Fig. 1A) [6]. While cryptic RNAs including *STMN2*-CE and *UNC13A*-CE have been detected in postmortem brain tissue [6], they have yet to be detected in biofluids, hindering their application for biomarker development. Perhaps most pertinent to biomarker development, consequently, are the cryptic transcripts that generate *de novo* proteins.

**Fig. 1:**
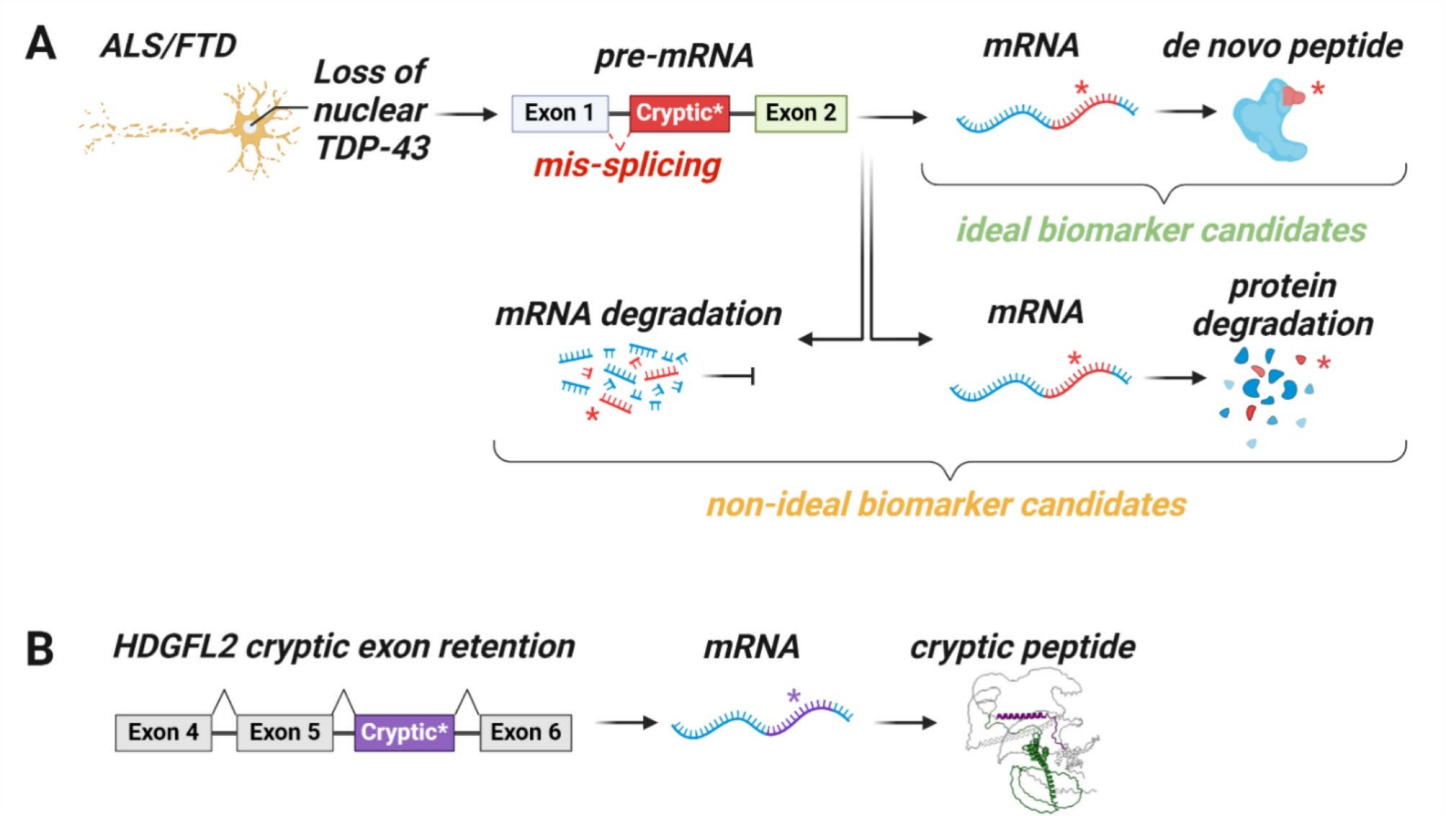
Inclusion of an in-frame cryptic exon within a mature RNA transcript can generate *de novo* cryptic peptides, such as HDGFL2-CE. (**A**) In response to TDP-43 nuclear depletion, transcripts can be misspliced to include a cryptic exon, disrupting the transcript and resulting in its degradation either at the RNA or protein level. Therefore, these targets are not viable for biomarkers. (**B**) In some cases, the cryptic exon can be incorporated in-frame, yielding a cryptic peptide, such as in HDGFL2, where a cryptic exon is incorporated in-frame between exons 5 and 6 in the mature transcript.

Seddighi et al. recently generated an atlas of CEs utilizing TDP-43-depleted human induced pluripotent stem cell (iPSC)-derived neurons to model nuclear TDP-43 loss of function. Notably, some CEs were found to interact with ribosomes, suggesting there may be active translation of these non-conserved sequence-retaining transcripts. Indeed, by combining transcriptomics with proteomics, they identified 65 cryptic peptides, more than half of which were predicted to incorporate an in-frame CE [7]. Therefore, Seddighi and colleagues developed antibodies for two such CE-containing peptides, HDGFL2-CE and MYO18A-CE. The hepatoma derived growth factor 2 (HDGFL2), a histone-binding protein that regulates chromatin accessibility and recruits regulatory factors to assist in DNA damage repair, is ubiquitously expressed throughout the central nervous system (CNS) [7]. When TDP-43 becomes dysfunctional, an in-frame CE is incorporated between exons 5 and 6 of the mature *HDGFL2* transcript, thereby producing HDGFL2-CE, a stable cryptic peptide (Fig. 1B) [7]. The second CE-containing peptide, Myosin XVIIIA (MYO18A), is a cytoskeletal protein moderately expressed in the CNS that modulates cell structure and migration [7]. Both *HDGFL2* and *MYO18A* CE-containing transcripts were found to be significantly elevated in postmortem frontal cortex tissues from FTLD-TDP patients, and their cryptic peptides were detected in cerebrospinal fluid (CSF) from patients with ALS or FTD, suggesting these cryptic peptides and others may serve as stable fluid biomarkers of TDP-43 dysfunction [7].

Similarly to the above-mentioned work, Irwin and colleagues sifted through RNA sequencing datasets from TDP-43-depleted HeLa cells and iPSC-derived motor neurons, likewise identifying *HDGFL2* transcripts harboring an in-frame CE [8]. Using a novel anti-HDGFL2-CE antibody and postmortem motor cortex and hippocampal tissues from ALS and FTLD-TDP cases, Irwin et al. ascertained that HDGFL2-CE was specifically detected in neurons depleted of nuclear TDP-43. Towards detecting HDGFL2-CE in biofluids, they developed a HDGFL2-CE immunoassay allowing them to measure HDGFL2-CE in CSF and plasma. Compared to controls, CSF HDGFL2-CE was higher in patients with sporadic ALS and in presymptomatic and symptomatic *C9orf72* repeat expansion carriers [8]. These findings are indeed encouraging, but as with all biomarkers, will require validation using larger cohorts and rigorous analyses.

To establish if HDGFL2-CE abundance can be used to gauge TDP-43 pathology and dysfunction, Calliari et al. investigated whether HDGFL2-CE is preferentially expressed in neuroanatomical regions with TDP-43 proteinopathy. To this end, they availed well-characterized cohorts of FTLD-TDP and AD-TDP postmortem cases coupled with a novel HDGFL2-CE immunoassay [9]. Compared to controls, they observed significantly higher HDGFL2-CE in the frontal cortex and amygdala in FTLD-TDP cases, and in the amygdala of AD cases with TDP-43 pathology. Of importance, the presence of HDGFL2-CE distinguished cases with and without TDP-43 pathology with good to excellent discriminatory ability. Furthermore, both *HDGFL2*-CE transcripts and HDGFL2-CE proteins positively correlated with phosphorylated TDP-43, a pathological trait of TDP-43 proteinopathy [9]. These findings demonstrate that HDGFL2-CE is a sensitive reporter of TDP-43 pathology in the CNS, and corroborate the use of CSF HDGFL2-CE as a surrogate marker of TDP-43 pathology and dysfunction [9].

Given the present dearth of TDP-43-associated biomarkers, continued investigations on HDGFL2-CE and other cryptic peptides are warranted. Although Seddighi et al. availed proteomic analyses to identify cryptic peptides in CSF from ALS/FTD patients [7], more sensitive quantitative proteomic approaches are required to ascertain whether these cryptic peptides are elevated in ALS/FTD. Nevertheless, validating HDGFL2-CE as a biomarker for TDP-43 dysfunction would benefit from more practical methods, such as the use of highly specific and sensitive immunoassays. Such validation studies would also require the quantification of HDGFL2-CE concentrations in CSF or plasma from large, thoroughly-characterized cross-sectional and longitudinal cohorts with comprehensive clinical data and, ideally, autopsy-confirmed pathology. As optimized HDGFL2-CE assays become available, they are expected to enable the early detection of TDP-43 dysfunction in presymptomatic, prodromal, and clinical stages of disease, thereby facilitating the recruitment of participants in prevention and early treatment trials for therapies targeting aspects of TDP-43 pathophysiology. This notion is bolstered by the fact that Irwin et al. detected HDGFL2-CE in CSF from presymptomatic and symptomatic *C9orf72* mutation carriers [8]. Although the studies discussed here reveal HDGFL2 is misspliced upon TDP-43 dysfunction [7–9], HDGFL2 splicing may be modulated by other proteins, which could confound its use as a marker for TDP-43 pathology and dysfunction. As such, despite the strong correlation between pathological TDP-43 and HDGFL2-CE in postmortem tissues supporting its utility as a TDP-43 marker [9], coupling HDGFL2-CE with a panel of other cryptic peptides including MYO18A, AGRN, and CAMK2B [7] warrants consideration as it could improve our confidence in accurately detecting TDP-43 dysfunction. It is thus worth noting that detection methods such as nucleic acid linked immuno-sandwich assays (NULISA) permit the simultaneous measurement of multiple cryptic peptides [10]. In tandem with these efforts, alternative biomarkers for identifying individuals with TDP-43 pathology are emerging.

As the field further probes the implications of cryptic peptides in FTD and ALS, investigating TDP-43 dysfunction in other TDP-43 proteinopathies should be taken into account. For example, muscle biopsies of patients with inclusion body myositis exhibit TDP-43 aggregates, nuclear TDP-43 clearance, and the inclusion of CEs in mRNA transcripts, including *HDGFL2* [11]. Recent work has also identified TDP-43-mediated misspliced cryptic transcripts, such as *STMN2*, *UNC13A,* and *HDGFL2* in AD and LATE [9, 12–14] suggesting that cryptic peptides as markers of TDP-43 dysfunction are relevant not only to ALS, FTD, AD, and LATE, but also to other neurodegenerative disorders with mixed pathologies such as Lewy body dementia, chronic traumatic encephalopathy, and other AD-related dementias.

As we further examine the utility of HDGLF2-CE as a biomarker, the functions of HDGFL2-CE and other cryptic proteins should be elucidated. Seddighi et al. found that HDGFL2-CE alters the HDGFL2 interactome, with HDGFL2-CE displaying increased interactions with RNA-binding proteins and decreased interactions with cytoskeletal proteins, suggesting that HDGFL2-CE induces both toxic gains and losses-of-function and may thus influence disease onset and progression [7]. Deciphering the pathomechanisms through which cryptic exon inclusions in transcripts contribute to neurodegeneration will broaden our understanding of disease pathogenesis and may provide a more targeted approach in treating TDP-43 proteinopathies.

## Data Availability

Not applicable.

## Abbreviations

AD: Alzheimer’s disease
ALS: Amyotrophic lateral sclerosis
CE: Cryptic exon
CNS: Central nervous system
CSF: Cerebrospinal fluid
FTD: Frontotemporal dementia
FTLD: Frontotemporal lobar degeneration
HDGFL2: Hepatoma derived growth factor
iPSC: Induced pluripotent stem cell
LATE: Limbic-predominant age-related TDP-43 encephalopathy
MYO18A: Myosin XVIIIA
NULISA: Nucleic acid linked immuno-sandwich assay
TDP-43: TAR DNA-binding protein of 43 kDa
